# 2022 CVS Caremark DOAC non-medical switch: Patient ramifications and physician advocacy

**DOI:** 10.1016/j.ajpc.2024.100870

**Published:** 2024-10-03

**Authors:** Kushal D. Patel, Dharmesh Patel, Hunt Deison, Ryan Gough

**Affiliations:** a Memphis University School, Memphis, TN, USA; b Woodberry Associates, Washington, DC, USA; c Stern Cardiovascular Foundation, Memphis, TN, USA; d Partnership to Advance Cardiovascular Health, Washington, DC, USA

**June 2024**

Just before Thanksgiving in 2021, CVS Caremark, the largest pharmacy benefit manager (PBM) in the country, sent a letter to 150,000 patients who were prescribed apixaban, a direct oral anticoagulant medication – or DOAC – for the treatment of venous thromboembolism (VTE), otherwise known as blood clotting. CVS Caremark's letter detailed their decision to remove all DOACs from its 2022 formulary list except for rivaroxaban [1,2].

DOACs are widely used among Americans and two medications in this class – apixaban and rivaroxaban – were selected for the first round of negotiations in the Medicare Drug Price Negotiation Program mandated by 2022′s Inflation Reduction Act.

Despite the wishes of clinicians who had written the prescriptions, patients were forced to either transition to rivaroxaban or pay out-of-pocket the full the cost of apixaban. While CVS Caremark also covers warfarin, a traditional non-DOAC anticoagulant, warfarin is less safe and more demanding with many dietary and drug interactions and frequent dosage changes [1,3]. CVS Caremark's decision to remove apixaban from their formulary was particularly consequential for patients as apixaban and rivaroxaban are not merely interchangeable.

PBMs such as CVS Caremark serve as the middlemen in the pharmaceutical marketplace. They buy medicines from manufacturers at a discounted price and then sell them to pharmacies at a profit. Due to vertical integration, PBMs negotiate with pharmaceutical manufacturers and wholesalers to decide which medications will be included on an insurer's formulary and how medications are tiered. PBMs’ sudden decision to change the formulary and force stable patients off their current medication to another therapy based on arbitrary and non-transparent profit intentions is called non-medical switching, and it poses a serious risk to patients prescribed anticoagulants.

The American Society for Preventive Cardiology and the Partnership to Advance Cardiovascular Health conducted a national survey of 254 patients on blood thinners who were impacted by non-medical switching to comprehend the adverse effects the questionable utilization management tactic has on patients. Each anticoagulant is uniquely tailored to patients based on their diverse medical background, so switching a medication that is already working will often harm the patient [4].

**Figure:**
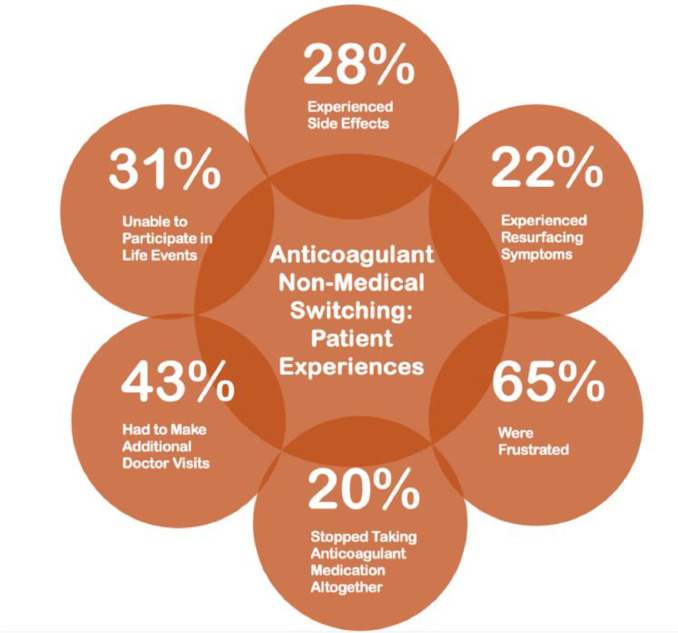

28 percent of patients reported side effects after being non-medically switched, and 22 percent reported resurfacing symptoms. For anticoagulant patients, the switch posed an even more serious risk, with 7 percent of patients suffering a heart attack and 4 patients suffering a stroke [4].

Patients’ lives should not be traded for profit by unnecessary, manipulative health plans. These medical tolls, coupled with 43 percent of switched patients making repeated doctor visits, 40 percent reporting additional lab tests, 17 percent having to visit the pharmacy again, and 5 percent being hospitalized all lead to frustration and non-adherence from patients. This exasperation causes one out of five patients to stop taking their anticoagulant medication altogether, putting them at even higher risk of clotting, in turn placing an increased burden on the health care system [4].

Even more concerning is how insurers force the switch. 94 percent of patients reported their insurers suddenly discontinued the medication, and 83 percent reported an increase of out-of-pocket expenses. This is particularly calamitous because 94 percent of patients depended on a blood thinner to go about their daily lives. Patients were either forced to cope with the switch and pay the price out-of-pocket, switch to a less effective option or simply forgo therapy altogether [4].

Still, after switching, patients struggled to enjoy themselves with 36 percent unable to continue hobbies, 31 percent unable to fully participate in life events and 23 percent unable to fulfill caregiving responsibilities. Through this pernicious system, the patient is cast aside with abject mental health consequences. 52 percent reported feeling helpless, 65 percent were frustrated, and 59 percent were left confused. Despite this harsh reality, 83 percent of patients understand that keeping their blood thinner is important and 96 percent placed high value on having the right one. Patients need their treatment decisions to stay between them and their doctors, and it is up to physicians to ensure the correct dynamic [4].

The American Society for Preventive Cardiology, the Partnership to Advance Cardiovascular Health and the American College of Cardiology all mobilized their medical experts to head an advocacy movement resisting CVS's decision. After numerous letters written to CVS Caremark and two observed instances of acutely adverse thrombotic events in North Carolina, CVS reversed their decision six months after implementing it [5].

The threat of non-medical switching for oral anticoagulants is especially pertinent now due to drug price negotiations under the Inflation Reduction Act. The Inflation Reduction Act mandates the Secretary of Health and Human Services to engage in price negotiations with pharmaceutical companies for specific drugs covered by Medicare Part D and Part B. On August 29, 2023, the government announced the first ten drugs which were picked for price negotiations, and, interestingly, two of the ten drugs were apixaban and rivaroxaban. After negotiations, when the final price is determined in September 2024, the prices of the two drugs will likely be different, creating an environment where PBMs may be tempted to switch patients to cheaper alternatives, potentially incurring a similar incidence to the 2022 rivaroxaban switch, which must serve as proof of the serious consequences of large-scale non-medical switching. More importantly, the cardiovascular community's rebuttal represents both a model for how physicians can protect themselves and their patients from non-medical switching as well as a call for future protection against the threat of large-scale switching [6].

Considering that 43 total drugs will have their prices re-negotiated through the IRA, non-medical switching will continue to blight countless medications. In a 2019 study of over 800 patients who experienced non-medical switching, the Alliance for Patient Access found that almost 60 percent of patients experienced a complication from the new medication, causing almost 40 percent to stop taking their medicine altogether [6,7].

Physician advocacy is the bedrock for preventing non-medical switching; the personal stories and unmediated experiences of doctors encourage more accurate and compelling advocacy efforts. Like in the rebuttal to CVS's rivaroxaban switch, physician advocacy roles are amplified in larger institutions such as the American Society for Preventive Cardiology and the American College of Cardiology. Within an organization of similarly oriented physicians and educators, physicians can write letters to PBMs and policymakers to educate them of the potentially life-threatening impact of non-medical switching. Policies should ensure that patients who are already on stable treatment cannot be deviated to an alternative medication without the approval of their physician, effectively protecting the patient from non-medical switching.

21 total bills that limit non-medical switching have already been passed throughout the United States. For example, Iowa passed House File 626 in May 2024, preventing insurers from switching a stable patient's prescribed drug without the written consent of the prescribing physician. The over 30 states that have not introduced any legislation against non-medical switching must follow the footsteps to give prescribing power back to the physician and set an example for the federal government [8].

## CRediT authorship contribution statement

**Kushal D. Patel:** Writing – original draft, Writing – review & editing. **Dharmesh Patel:** Writing – original draft, Writing – review & editing. **Hunt Deison:** Writing – review & editing. **Ryan Gough:** Supervision.

## Declaration of competing interest

The authors declare the following financial interests/personal relationships which may be considered as potential competing interests:

Dharmesh Patel, MD reports financial support was provided by BMS Pfizer Alliance. If there are other authors, they declare that they have no known competing financial interests or personal relationships that could have appeared to influence the work reported in this paper.

## References

[1] Waldron B. (2022). Nonmedical switching of anticoagulants: the patient impact when formulary exclusions limit drug choice. Res Pract Thromb Haemostasis.

[2] DrSJ Baum (2023). Non-medical switching an unmitigated threat to patient care. Am J Prev Cardiol.

[3] Julia S., James U. (2017). Direct oral anticoagulants: a quick guide. Eur Cardiol.

[4] Advocacy statements - American Society for Preventive Cardiology. Accessed June 11, 2024. https://www.aspconline.org/advocacy-statements.

[5] Craycroft-Andrews S., Smart C., Jones D., Dunn S., Jarnigan R., Morrison S.R. Pharmacy and therapeutics (P&T) committee. Published online 2022.

[6] Gulati M. (2023). President's page: the impact of the inflation reduction act on cardiovascular disease prevention. Am J Prev Cardiol.

[7] A study of the qualitative impact of non-medical switching. alliance for patient access; 2019. Accessed June 17, 2024. https://allianceforpatientaccess.org/a-study-of-the-qualitative-impact-of-non-medical-switching/.

[8] Nonmedical switching - enacted laws. Aimed alliance. Accessed June 20, 2024. https://aimedalliance.org/nonmedical-switching-enacted-laws/.

